# Identification of a Novel Missense *FBN2* Mutation in a Chinese Family with Congenital Contractural Arachnodactyly Using Exome Sequencing

**DOI:** 10.1371/journal.pone.0155908

**Published:** 2016-05-19

**Authors:** Hao Deng, Qian Lu, Hongbo Xu, Xiong Deng, Lamei Yuan, Zhijian Yang, Yi Guo, Qiongfen Lin, Jingjing Xiao, Liping Guan, Zhi Song

**Affiliations:** 1 Department of Neurology, the Third Xiangya Hospital, Central South University, Changsha, 410013, China; 2 Center for Experimental Medicine, the Third Xiangya Hospital, Central South University, Changsha, 410013, China; 3 Department of Medical Information, Information Security and Big Data Research Institute, Central South University, Changsha, 410013, China; 4 BGI-Shenzhen, Shenzhen, 518083, China; Huashan Hospital, Fudan University, CHINA

## Abstract

Congenital contractural arachnodactyly (CCA, OMIM 121050), also known as Beals-Hecht syndrome, is an autosomal dominant disorder of connective tissue. CCA is characterized by arachnodactyly, dolichostenomelia, pectus deformities, kyphoscoliosis, congenital contractures and a crumpled appearance of the helix of the ear. The aim of this study is to identify the genetic cause of a 4-generation Chinese family of Tujia ethnicity with congenital contractural arachnodactyly by exome sequencing. The clinical features of patients in this family are consistent with CCA. A novel missense mutation, c.3769T>C (p.C1257R), in the fibrillin 2 gene (*FBN2*) was identified responsible for the genetic cause of our family with CCA. The p.C1257R mutation occurs in the 19th calcium-binding epidermal growth factor-like (cbEGF) domain. The amino acid residue cysteine in this domain is conserved among different species. Our findings suggest that exome sequencing is a powerful tool to discover mutation(s) in CCA. Our results may also provide new insights into the cause and diagnosis of CCA, and may have implications for genetic counseling and clinical management.

## Introduction

Congenital contractural arachnodactyly (CCA, OMIM 121050), also known as Beals-Hecht syndrome, is an autosomal dominant disorder of connective tissue. CCA is phenotypically related to but genetically distinct from Marfan syndrome (MFS) [1]. It was first introduced by Rodney Beals and Frederick Hechet in 1971. It is characterized by contractures, arachnodactyly, narrow body habitus, elongated limbs, chest wall deformities, scoliosis, muscular hypoplasia and crumpled ears [2,3]. It can be divided into classical CCA and severe/lethal CCA. Patients with severe/lethal CCA show cardiac abnormalities (mitral valve prolapse, atrial septal defect, ventricular septal defect and aortic hypoplasia) and gastrointestinal anomalies (duodenal atresia, intestinal malrotation), in addition to anomalies presented in classical CCA [4]. The estimated incidence of CCA is not clear but seems to be less frequent than that of MFS with the incidence of 1:10,000 [5]. CCA showed no geographic or ethnic predilection. Although it has been described mainly in Caucasian families of European origin, CCA has also been reported in Japanese, people of Indian descent, African Americans, African Blacks [6], and a Chinese family [7]. The *FBN2* gene, discovered during the cloning of the *FBN1* gene, was mapped to chromosome 5q23-q31 and was linked to CCA [8].

The main purpose of this study was to identify the gene responsible for a 4-generation Chinese family with CCA that is characterized by arachnodactyly, camptodactyly, kyphoscoliosis and large joint contracture. We found a heterozygous c.3769T>C transition (p.C1257R), which co-segregates with patients in this family and are absent in normal controls, in the *FBN2* gene. The missense mutation disrupts the overall integrity of the FBN2 protein. Our data indicate that it is a pathogenic mutation.

## Materials and Methods

### Participators and clinical evaluation

**Fig 1:**
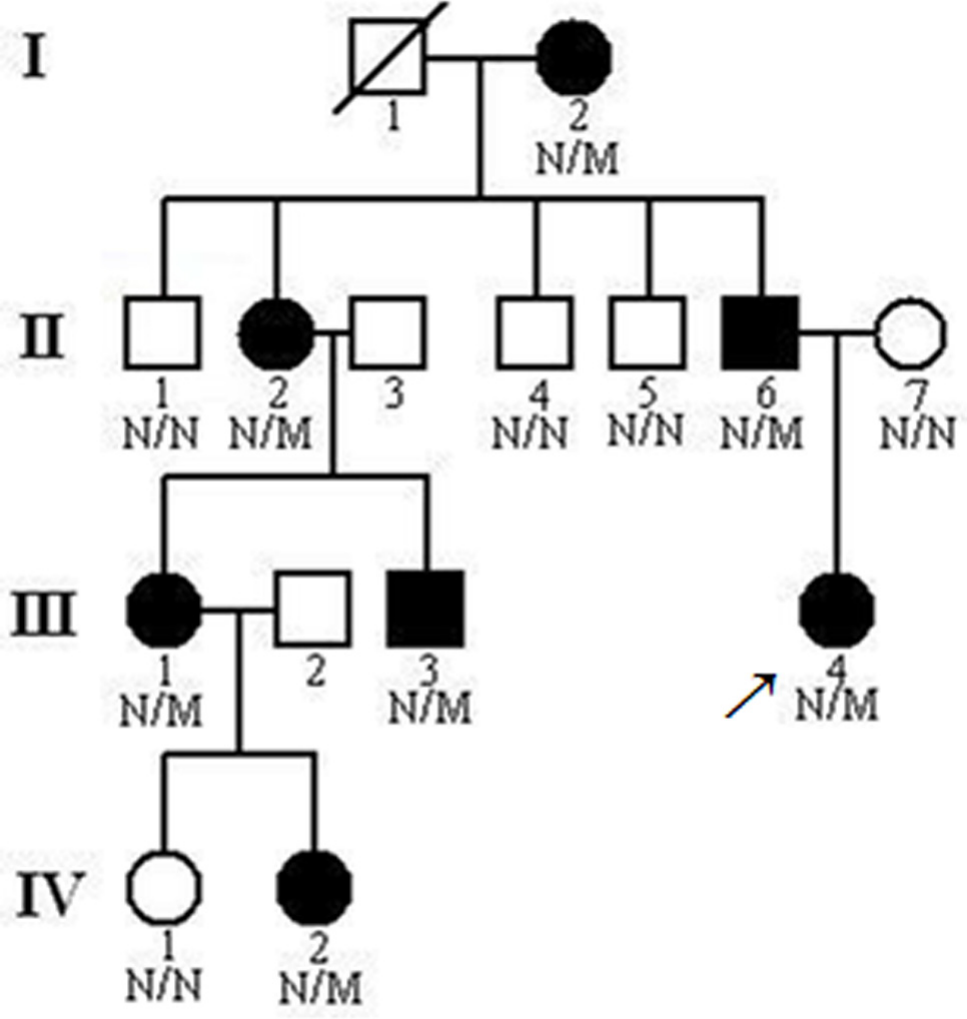
Pedigree of the family with congenital contractural arachnodactyly showing affected cases (fully shaded). N, Normal; M, the *FBN2* c.3769T>C (p.C1257R) mutation. The arrow indicates the proband.

A 4-generation, 15-member Chinese family of Tujia ethnicity with familial CCA was recruited from the Third Xiangya Hospital, Central South University (Fig 1). Blood samples were collected from 14 members of the family, including 7 affected individuals (Ι:2, II:2, II:6, III:1, III:3, III:4 and IV:2,) and 7 unaffected members (II:1, II:3, II:4, II:5, II:7, III:2, and IV:1) (Fig 1). Blood samples were also collected from 100 unrelated normal controls (male/female: 50/50, age 45.7±6.5 years). Written informed consent was obtained from all subjects, and this study was approval from the Ethics Committee of the Third Xiangya Hospital, Central South University, China.

### Exome capture and sequencing

Genomic DNA was extracted from blood samples using standard phenol-chloroform extraction method [9]. Exome capture was performed in the proband (III:4), by BGI-Shenzhen using NimbleGen SeqCap EZ Human Exome Library v2.0 (Roche NimbleGen, Inc., Madison, WI, USA), and sequencing was performed using a HiSeq 2000 platform (Illumina, San Diego, CA, USA). All steps were performed according to the manufacturer’s instructions [10]. The enriched library targeting the exome was sequenced on the HiSeq 2000 platform to get paired-end reads with a read length of 90-bp [11]. Exome sequencing depth of 68.01× were obtained to provide sufficient depth to accurately call variants at 99.17% of each targeted exome.

### Read mapping and variant analysis

The clean reads of each individual were aligned to the human reference genome (UCSC Build 37.1, hg19) using Burrows-Wheeler transform (BWA; Cambridge, UK) and made variant calls with Genome Analysis Tool Kit (version 2.1), and completed functional annotation of the variants with BGI in-house script [12,13]. Alignment of the sequences from one affected individual of the family was performed using SOAPaligner (soap2.21) after the duplicated reads were deleted, and SNPs were called using SOAPsnp (1.05) set with the default parameters [14]. Insertions or deletions (indels) affecting coding sequence or splicing sites were identified [15]. The thresholds for calling SNPs and short insertions or deletions included the number of unique mapped reads supporting a SNP ≥4 and the consensus quality score ≥20. The quality score is a Phred score, generated by the program SOAPsnp1.05, quality score 20 represents 99% accuracy of a base call. All candidate mutations in the subject were filtered against the single nucleotide polymorphism databases (dbSNP build 137, http://www.ncbi.nlm.nih.gov/projects/SNP/snp_summary.cgi), ethnic Han Chinese individuals from Beijing available in the 1000 Genomes Project (1000genomes release_20100804, http://www.1000genomes.org/), HapMap (2010–08_phaseII+III, http://hapmap.ncbi.nlm.nih.gov/) and YanHuang (http://yh.genomics.org.cn/) project [16].

### Mutation validation

Sanger sequencing was performed to confirm the presence and identity of potential disease-causing variants with ABI3500 sequencer (Applied Biosystems Inc., Foster City, CA, USA). PCR amplification was conducted as described previously [17], and the primer sequences were as follows: 5′-ATACCTGCACACGATCTCCC-3′ and 5′-AAGCAGACCTGACAATGTGG-3′.

### Bioinformatics analysis of the mutation

The Basic Local Alignment Search Tool (http://blast.st-va.ncbi.nlm.nih.gov/Blast.cgi) was used to perform multiple sequence alignment. Online tools, including MutationTaster (http://www.mutationtaster.org/), Polymorphism Phenotyping version 2 (PolyPhen-2, http://genetics.bwh.harvard.edu/pph2/) and SIFT (http://sift.jcvi.org/, scores less than 0.05 are deleterious), were used to evaluate the possible effects of amino acid substitution on protein structure and function in terms of chemical change, sequence conservation, and likelihood of pathogenicity [18,19,20].

## Results

### Clinical findings

**Fig 2:**
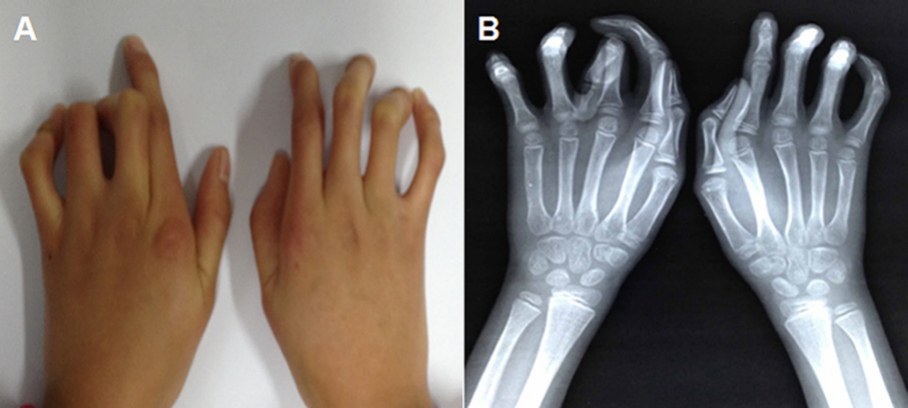
**(A) The phenotype and (B) X-ray images of hands from an affected member (IV:2) of the family**.

Clinical features of the seven patients in this family who participated in this study are shown in Table 1. The proband (III:4), a 11 year old girl, was noted at birth to have contractures of the fingers without any prenatal complication, and had some improvement of her contractures with age. She had normal mental and motor development, and the results of blood and urine examinations were normal. On physical examination, all seven patients (Ι:2, II:2, II:6, III:1, III:3, III:4 and IV:2) showed arachnodactyly and camptodactyly (Fig 2), two patients (II:6, III:3) showed kyphoscoliosis and large joint contracture, and one patient (III:6) had cardiovascular abnormalities. There was no evidence of tall stature, crumpled ears or ocular complication. Compare to female patients, male patients experienced more severe phenotypes, such as kyphoscoliosis and cardiovascular complications, which have not been reported before.

**Table 1:** Clinical and genetic data of 7 patients with *FBN2* c.3769T>C (p.C1257R) mutation.

Subject	I:2	II:2	II:6	III:1	III:3	III:4	IV:2
**Gender**	Female	Female	Male	Female	Male	Female	Female
**Age (years)**	85	58	44	39	30	11	10
**Genotype**	Heterozygote	Heterozygote	Heterozygote	Heterozygote	Heterozygote	Heterozygote	Heterozygote
**Tall stature**	-	-	-	-	-	-	-
**Crumpled ear**	-	-	-	-	-	-	-
**Arachnodactyly**	+	+	+	+	+	+	+
**Camptodactyly**	+	+	+	+	+	+	+
**Large joint contracture**	-	-	+	-	+	-	-
**Kyphoscoliosis**	-	-	+	-	+	-	-
**Muscle hypoplasia**	-	-	-	-	-	-	-
**Cardiovascular complication**	-	-	+	-	-	-	-
**Ocular complication**	-	-	-	-	-	-	-

+, present; -, absent

### Mutation screening

We performed exome sequencing of the proband (III:4, Fig 1) in the Chinese family with CCA, and 6.74 billion bases of 90-bp paired-end read sequence were generated. Among the 6.74 billion bases, 6.40 billion (94.85%) passed the quality assessment, 6.22 billion (92.22%) were aligned to the human reference sequence, and 4.27 billion bases (63.35%) were mapped to the targeted bases with a mean coverage of 68.01-fold. A total of 111,167 genetic variants, including 14,779 non-synonymous variants, were identified in either the coding regions or the splice sites. We excluded known variants identified in dbSNP137, 1000 human genomes project, HapMap, and YanHuang. After this, we reduced the number of candidate genes by more than 93.17%. The sequencing data of our study was deposited in NCBI Sequence Read Archive (SRA) database (study accession number: SRP071315).

**Fig 3:**
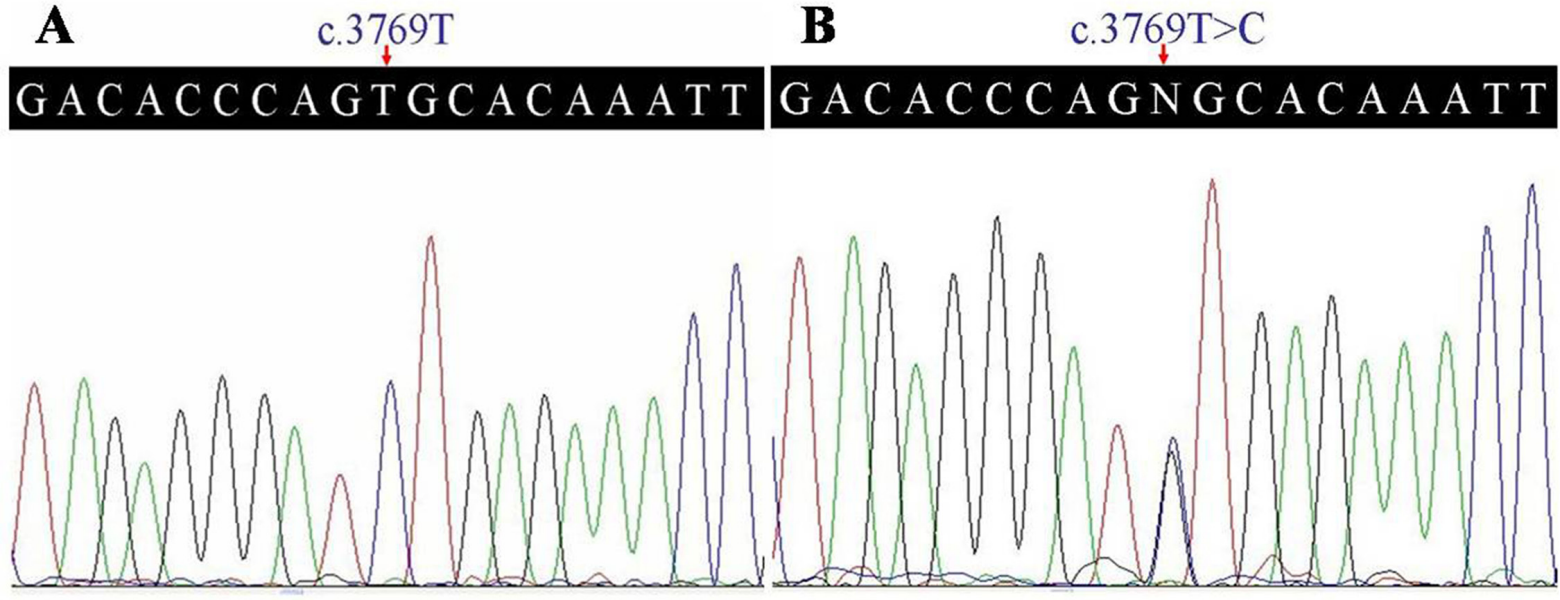
Sequencing analysis of p.C1257R mutation in the *FBN2* gene (DNA). (A) Unaffected member (II:7) of the family. (B) Heterozygous p.C1257R mutation patient (III:4).

A prioritization scheme was applied to identify the pathogenic mutation in the proband. After validation by Sanger sequencing, a c.3769T>C (p.C1257R) mutation in the *FBN2* gene was identified in the proband (Fig 3). Two male patients (II:6, III:3) and five female patients (Ι:2, ΙΙ:2, ΙΙΙ:1, ΙΙΙ:4, IV:2) in the family were subsequently found to carry the same heterozygote mutation. The variant co-segregated with disease phenotype in this family, and was absent in unaffected individuals in this family and in 100 ethnicity-matched unrelated controls.

### Bioinformatics analysis of the mutation

**Fig 4:**
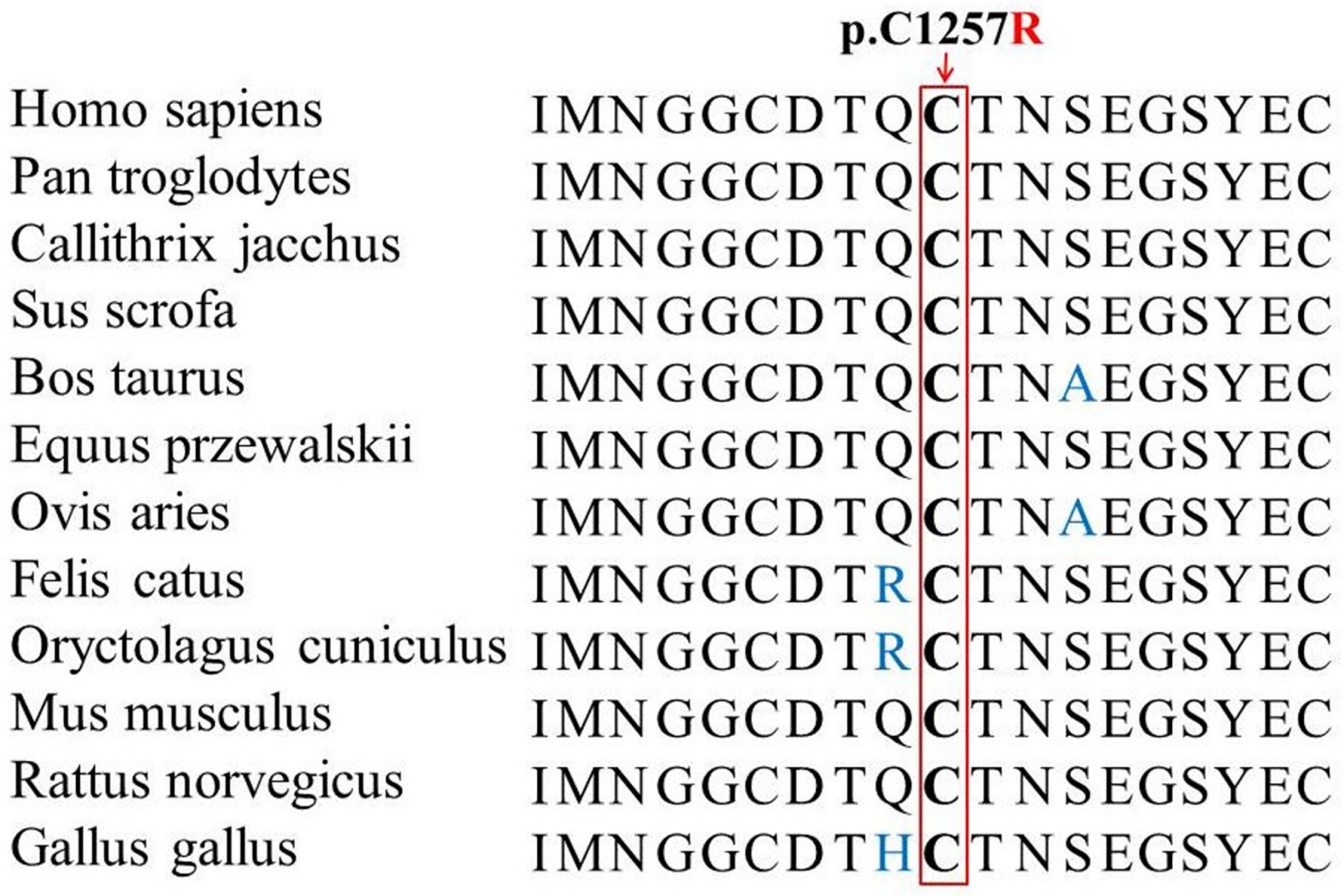
Conservation analysis of *FBN2* p.C1257 amino acid residue.

The cysteine at position 1257 is phylogenetically conserved among various species (Fig 4). MutationTaster predicted that the mutation was disease-causing with a probability value close to 1. PolyPhen-2 analysis produced a score of 0.991 on the HumVar database (sensitivity, 0.50; specificity, 0.95), which is predicted to be probably damaging. The SIFT prediction revealed a score of 0.00, indicating that the substitution is predicted to affect protein function. Our data indicated that the variant c.3769T>C (p.C1257R) in the *FBN2* gene was likely deleterious and was the disease-causing mutation for CCA in our family.

## Discussion

CCA is a well-characterized autosomal dominant disorder with variability in the clinical expression and intragenic heterogeneity [21]. CCA is phenotypically related to MFS, including tall stature, marfanoid habitus, arachnodactyly, camptodactyly, kyphoscoliosis and pectus excavatum [22]. However, CCA patients have crumpled appearance of ear helix and multiple joint contractures (especially elbow, knee and finger joints), and usually do not produce the ocular and life-threatening cardiovascular complications observed in MFS [23,24]. CCA can be distinguished from MFS genetically because CCA is caused by the mutation of the *FBN2* gene, whereas MFS is caused by the *FBN1* gene defects. Fibrillin 1 and fibrillin 2 are major structural components of the extracellular microfibrils with an average diameter of 10 nm, and they have virtually super imposable structures, with almost every domain encoded by a separate exon [25]. Fibrillin is a component of the extracellular matrix microfibrils, which play an essential role in the formation of elastic fiber and the deposition of tropoelastin, and perform anchoring functions in some tissues. Fibrillin 1 is involved in tissue rigidity while fibrillin 2 is associated with tissue elasticity [26,27].

The *FBN2* gene (OMIM 612570) located at chromosome 5q23-31, consists of 65 exons and encodes a 2,912-amino acid protein [28]. It is characterized by long introns taking up the first half of the gene and the largest intron is 54,341 bps (between coding exons 5 and 6), and densely packed exons in the second half. The evolutionary conservation of intron length in the *FBN2* gene across the mammals indicates that the introns may have some function in regulating gene expression. The large introns may contain promoters and/or enhancer elements for non-coding RNAs, or influence the rate of production of mature mRNA [29]. At least 48 mutations in the *FBN2* gene have been found to be responsible for CCA. These mutations vary from point mutations to gross deletions. Twenty-nine missense mutations, 13 splice-site mutations, a nonsense mutation, a small insertion, a small deletion, a gross insertion and 2 gross deletions are described in Human Gene Mutation Database (http://www.hgmd.cf.ac.uk/ac/index.php). The majority of *FBN2* mutations associated with CCA occur in a rather limited region in exons 23–35, a cbEGF rich region of fibrillin 2, similar to where severe MFS cluster in *FBN1*, between exons 23 and 34, the so-called "neonatal region", while only two are recorded near the N terminus, in exons 8 and 17 [24,30].

Exome sequencing revealed a T>C substitution at nucleotide 3769 (p.C1257R) in the *FBN2* gene of the proband. The mutation is located in exon 29, which encodes a calcium-binding epidermal growth factor-like (cbEGF) domain. The mutation alters the amino acid 1257 from cysteine to arginine, and is predicted to disrupt the secondary structure of the domain. This position is highly conserved among many different species, suggesting it is important for the stability and function of the protein (Fig 4) [31]. The abnormal fibrillin 2 may perturb the assembly of fibrillin into multimeric beaded microfibrils, normal function or stability of the microfibrils, and disrupt elastic fibrillogenesis [32,33]. Patients in this family have classical phenotype of CCA, without any evidence of tall stature and crumpled ears. One unique feature of our CCA family is that male patients present with more severe phenotype, such as large joint contracture, kyphoscoliosis and cardiovascular complications, which does not showed in five female patients. Such difference between genders may be related to genetic background, epigenetic and environmental factors, including the difference in the secretion of sex hormone and growth hormone, and a combination of subtle anatomical and physiological variations between males and females, etc. Further studies to use appropriate genetic-deficient animal models, detect the sex and growth hormone level in CCA patients, and analyze large sample of patients with CCA may facilitate a more thorough understanding of these differences in this disease.

Mouse models with spontaneous mutations, chemical mutagenesis, radiation-induced mutations and knockout mutant in the *Fbn2* gene have been described in the literature. Current mouse models recapitulate some, but not all aspects of the phenotype in human. The typical phenotype in homozygous mutation mouse models is syndactyly, and other manifestations, including transitory neonatal contractures, deafness and muscle weakness. No external ear deformities, arachnodactyly or spine anomalies have been reported, suggesting species-specific differences in development [34].

In summary, our data show that the novel missense mutation p.C1257R in the *FBN2* gene is the genetic cause of a Chinese Tujia family with CCA. Exome sequencing provides a highly efficient and cost-effective approach for identifying disease-causing gene of CCA while exclude gene(s) responsible for phenotype-similar disorders, such as MFS. Our findings may provide new insights into the cause of CCA and may have implication in genetic counseling for families with CCA.
